# INTERPROFESSIONAL SKILLS AND CULTURAL AWARENESS OF STUDENTS CARING FOR OLDER ADULTS WITH MULTIPLE CHRONIC CONDITIONS

**DOI:** 10.1093/geroni/igz038.2799

**Published:** 2019-11-08

**Authors:** Kathryn M Daniel, Mary Lou Bond

**Affiliations:** University of Texas at Arlington, Arlington, Texas, United States

## Abstract

Based on rapidly increasing numbers of older adults and growing populations of culturally diverse citizens, we developed and provided an interprofessional education program for graduate nursing and social work students that focused on the delivery of care to older adults and veterans with multiple chronic conditions. The students participated in multiple activities together over a year long period. Activities included an introductory IPE day with all students together and simulated patient cases that had required roles for the students’ specialties. Knowledge and skills of interprofessional collaborative practice were measured with the Interprofessional Self Assessment Competencies (IPEC) and Assessment of Interprofessional Team Collaboration Scale (AITCS). We also measured attitudes toward cultural differences using the Cultural Awareness Scale (CAS). The entire project was repeated a second year with a second cohort of students. Overall, our students were better able to understand and work collaboratively with other health care professionals after participating in this program. Regarding cultural awareness, both NP and SW students were more aware of and comfortable with cultural differences after participating in the program. After one year of experience, we wanted to know more about our students’ skills and attitudes. Focus groups were added to the design to dive deeper into the students’ perceptions about which activities were most impactful and recommendations for future IPE activities. This paper will describe these data and implications for future planning of more effective interprofessional and cultural programming for students caring for older adults and veterans.

